# Novel canine circovirus strains from Thailand: Evidence for genetic recombination

**DOI:** 10.1038/s41598-018-25936-1

**Published:** 2018-05-14

**Authors:** Chutchai Piewbang, Wendy K. Jo, Christina Puff, Erhard van der Vries, Sawang Kesdangsakonwut, Anudep Rungsipipat, Jochen Kruppa, Klaus Jung, Wolfgang Baumgärtner, Somporn Techangamsuwan, Martin Ludlow, Albert D. M. E. Osterhaus

**Affiliations:** 1 Department of Pathology, Faculty of Veterinary Science, Chulalongkorn University, Pathumwan, Bangkok, 10330 Thailand; 2 Research Center for Emerging Infections and Zoonoses, University of Veterinary Medicine (TiHo-RIZ), Bünteweg 17, Hannover, 30559 Germany; 3 Department of Pathology, University of Veterinary Medicine, Bünteweg 17, Hannover, 30559 Germany; 4 Institute for Animal Breeding and Genetics, University of Veterinary Medicine, Bünteweg 17p, Hannover, 30559 Germany; 5 STAR Diagnosis and Monitoring of Animal Pathogen, Faculty of Veterinary Science, Chulalongkorn University, Pathumwan, Bangkok, 10330 Thailand

## Abstract

Canine circoviruses (CanineCV’s), belonging to the genus *Circovirus* of the Circoviridae family, were detected by next generation sequencing in samples from Thai dogs with respiratory symptoms. Genetic characterization and phylogenetic analysis of nearly complete CanineCV genomes suggested that natural recombination had occurred among different lineages of CanineCV’s. Similarity plot and bootscaning analyses indicated that American and Chinese viruses had served as major and minor parental viruses, respectively. Positions of recombination breakpoints were estimated using maximum-likelihood frameworks with statistical significant testing. The putative recombination event was located in the Replicase gene, intersecting with open reading frame-3. Analysis of nucleotide changes confirmed the origin of the recombination event. This is the first description of naturally occurring recombinant CanineCV’s that have resulted in the circulation of newly emerging CanineCV lineages.

## Introduction

Circoviruses are non-enveloped DNA viruses belonging to the genus *Circovirus* of the family *Circoviridae*, and contain a small circular, single stranded ~2 kb DNA genome^1^. This genus harbors viruses that infect domestic and wildlife animal species, including porcine circoviruses (PCV-1 and -2), canary circovirus (CaCV) and beak and feather disease virus (BFDV) of birds^2–5^. Next generation sequencing (NGS) has recently allowed the discovery of additional mammalian circoviruses including PCV-3 in pigs^6^ and canine circovirus-1 (CanineCV-1) in dogs^1^. Infection with CanineCV-1, recently re-classified as ‘CanineCV’^7^, has been associated with several disease entities accompanied by manifestations like vasculitis, hemorrhages, thrombocytopenia, neutropenia and diarrhea^2,7–9^, although it is also detected in healthy dogs^1,8,10^. Interestingly, dogs infected with CanineCV are often co-infected with other enteric or respiratory pathogens^2,7,10,11^. Therefore, the pathogenic role of CanineCV is not clear. To date, CanineCV-infected dogs have been documented in several countries including the USA^1,8,11^, Italy^9^, Germany^7^, China (GenBank accession number: KT946839) and Taiwan^2^.

Besides genetic mutation, recombination is a driver of circovirus evolution, as several previous studies have shown genetic recombination within the *Circoviridae* family. These include BFDV^12^, Torque teno virus^13^ and PCV-2^14–17^, and have contributed to genetic diversity of these viruses. Types of recombination mechanisms include homologous recombination in which homologous sequences are exchanged, non-homologous recombination in which genome regions are rearranged, deleted, duplicated or inserted into the host genome and reassortment in which whole genome components of the virus get exchanged between strains and species^18^. In homologous recombination, any interruption during replication, such as the encounter of strand break sites or clashes between replication and transcription complexes, can create temporary detachment of the replication enzyme. The enzyme can then use another similar template to reinitiate replication, thus generating a recombinant genome^18^. Thus far, genetic recombination of CanineCV genomes has not been documented. In the present study, we have molecularly characterized CanineCV strains from Thai dogs by next generation sequencing with special emphasis on the occurrence of genetic recombination.

## Results

### Routine virological investigations of Thai dogs

Samples from three autopsied Thai dogs that had exhibited respiratory symptoms and had been subjected to routine diagnostics were selected for further investigation. This included multiplex PCR screening for a panel of common canine respiratory viruses, which showed the presence of canine influenza virus (CIV) and canine respiratory coronavirus (CRCoV) in the nasal swab of dog no. 14P105D and canine parainfluenza (CPIV) in the oral swab of dog no. 14P112N (Table 1). No viruses had been detected using the multiplex PCR screen in the lungs of these two dogs or in samples from dog no. 15P061D. In addition, no viruses had been detected upon screening of respiratory swabs collected from twenty other dogs with respiratory problems of varying severity, using multiplex PCR or pan-PCRs for corona-, paramyxo- and herpesviruses (Table 1). In addition, no indications for the presence of canine parvovirus (CPV) or canine coronavirus (CCV) were found in intestinal swabs taken from the three autopsied dogs (Nos 14P105D, 14P112N, 15P061D) using an antigen detection kit.

**Table 1:** Overview of CanineCV-positive samples.

Sample No.	Year	Age	Sample	CanineCV	Other viruses	Accession no.
15P105D	2014	7 m	Lung	+	CIV^b^, CRCoV^b^	MG737378
			Liver	+		
			Kidney	NSA		
			Brain	−		
			Tonsil	+		
			Lymph node	+		
			Others^a^	N/A		
14P112N	2014	1 m	Lung	+	CPIV^c^	MG737386
			Liver	+		
			Kidney	NSA		
			Brain	−		
			Tonsil	+		
			Lymph node	+		
			Others^a^	N/A		
15P106D	2015	4 y	Lung	+		MG737379
			Liver	+		
			Kidney	+		
			Brain	−		
			Tonsil	NSA		
			Lymph node	+		
			Others^a^	N/A		
CP28	2014	9 m	Swab	+		MG737380
CP134	2014	3 m	Swab	+		MG737381
CP144	2015	2 Y	Swab	+		MG737382
CP182	2015	3 m	Swab	+		MG737383
CP188	2014	2 m	Swab	+		MG737384
CP191	2014	6 m	Swab	+		MG737385

^a^Other tissues included small intestine, gall bladder, heart and thymus; ^b^Virus detection in nasal swab only; ^c^Virus detection in oral swab only.

Abbreviation: m, month; y, year; N/A, no data available; NSA, no sample available.

### Detection of CanineCV by NGS, conventional PCRs and ***in situ*** hybridization

To identify other viral pathogens associated with respiratory disease, lung samples of dog no.14P105D and 14P112N were processed for NGS. Over 900,000 trimmed reads were obtained from individual samples, of which approximately 853 (64x coverage) and 18 (0.7x coverage) reads were detected with highest homology to CanineCV in samples originating from dog no. 14P105D and dog no. 14P112N, respectively. Only partial CanineCV sequences were obtained by NGS analysis. Additional sequences were obtained using conventional PCR strategies based on primers designed from the NGS output sequences. The resulting consensus sequences represented ~92% of the complete CanineCV genome and confirmed the detection of this virus in both dogs, with the two strains named CanineCV_14P105D/TH2016 (accession no. MG737378) and CanineCV_14P112N/TH2016 (accession no. MG737386), respectively. Moreover, conventional PCR specific for CanineCV showed positive results in six out of the 20 nasal swabs, namely those coming from CP_28, CP_134, CP_144, CP_182, CP_188 and CP_191, and lung sample of dog no. 15P061D. The genomes of CanineCV in the swab samples were named CanineCV_CP28/TH2016 (MG737380), CanineCV_CP134/TH2016 (MG737381), CanineCV_CP144/TH2016 (MG737382), CanineCV_CP182/TH2016 (MG737383), CanineCV_CP188/TH2016 (MG737384), CanineCV_CP191/TH2016 (MG737385) and in the additional lung tissue sample, CanineCV_15P061D/TH2016 (MG737379).

**Figure 1:**
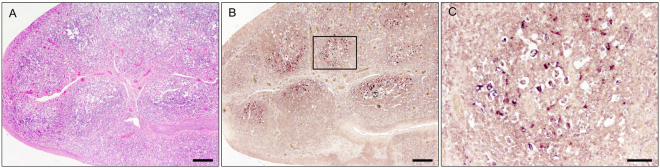
Histology of hyperplasia tonsillitis (**A**) and *in situ* hybridization (**B**,**C**) of CanineCV specific probe of dog no 14P105D TH/2016 showing strong positive signals in lymphoid follicles of the tonsil (**B**), located in the cytoplasm of lymphoid cells (**C**). The lymphoid follicle shown at higher magnification in (**C**) is indicated by a box in (**B**). Bar: A,B = 200 µm; C = 50 µm.

To investigate the organ distribution of CanineCV, CanineCV-specific PCR successfully detected the virus genome in lung, liver and tracheobronchial lymph node in the three dogs from which tissue samples were collected (Table 1). CanineCV nucleic acid was also detected in hyperplastic tonsils of dog nos. 14P015D and 14P112N, whereas it was detected in kidneys of dog 15P061D. Brain tissues of all dogs were negative (Table 1). *In situ* hybridization revealed single weakly positive cells in a lymph node and strongly positive cells within the follicle center of the tonsils from one dog (14P105D) (Fig. 1A–C). All other tissue samples including lung, liver, spleen, intestine and kidney from all three animals were negative for CanineCV by *in situ* hybridization.

### Genome organization, phylogenetic and recombination analyses of Thai CanineCV’s

**Figure 2:**
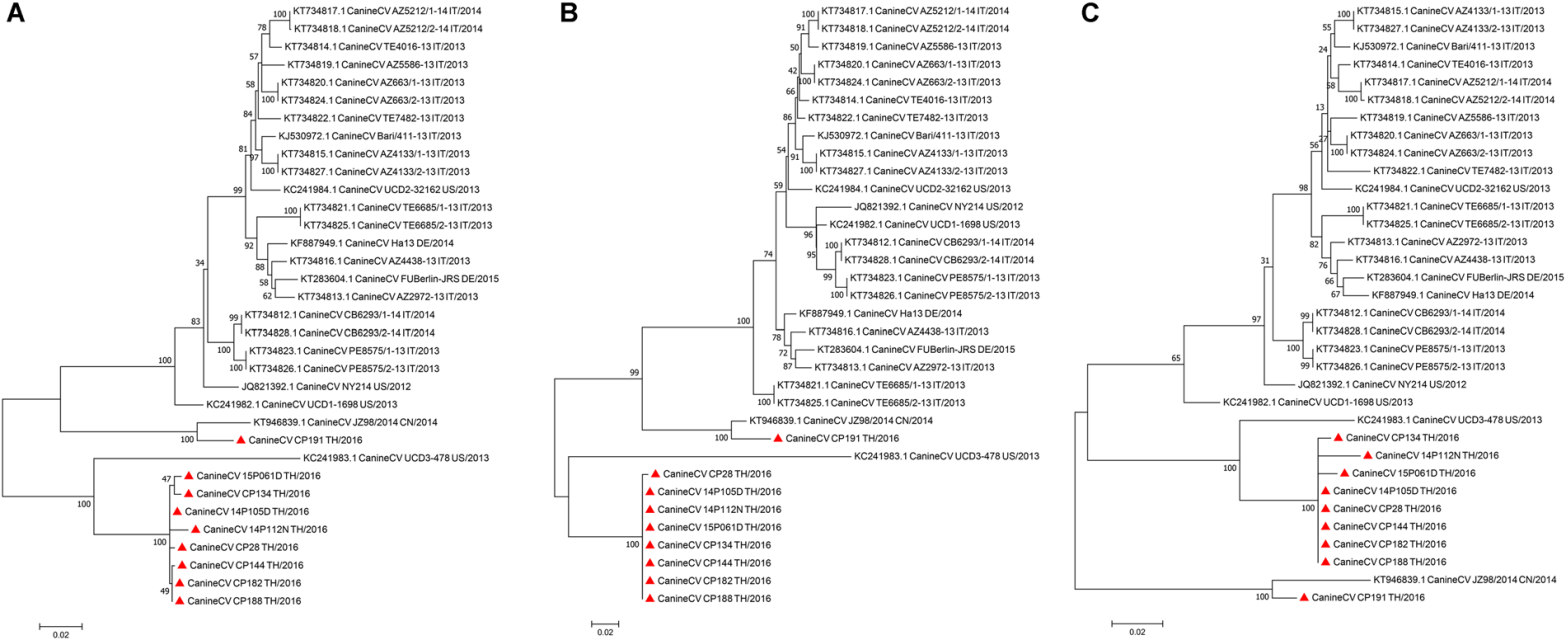
Phylogenetic tree of CanineCVs shows that strains detected in Thailand (red triangles) were mostly clustered with CanineCV UCD3-478; however, the CanineCV CP191 TH/2016 was clustered with CanineCV JZ98/2014 (**A**). The phylogenetic tree of individual Rep (**B**) and Cap genes (**C**) of CanineCV genomes were also constructed and suggested the CanineCV circulating in Thailand were divergent and revealed discrepant patterns among genes by showing distinct and divergent homologous clade of Rep gene, compared to Cap gene.

As expected, the coding sequence of all CanineCV’s TH/2016 contained 2 main open reading frames (ORFs), which encode a putative replication associated (Rep) and capsid (Cap) proteins. The Rep gene, nt 1-912, encoded 303 amino acids and the Cap gene, nt 1,116–1,928, encoded 270 amino acids. The origin of replication site (*ori*) was located at nt sites 2013–2020 (AGTATTAC). Moreover, one additional ORF, ORF3, located at nt 470–889 in the antisense of ORF1 was also identified. Pairwise distances revealed that the CanineCV’s TH/2016 differed by 10.5–16.2% from the USA-origin CanineCV’s UCD3-478 and Italy-origin AZ5586-13. Interestingly, most of the CanineCV’s detected in this study were most closely related to CanineCV_UCD3-478. However, CanineCV CP_191/TH2016 was found to be most closely related to the China-origin JZ98/2014 strain within another lineage (Supplementary Table S1). Phylogenetic analysis confirmed that the majority of the CanineCV’s TH/2016 were divergent to most CanineCV’s and were most closely related to CanineCV UCD3-478 (Fig. 2A). The individual Rep and Cap genes were used to construct additional phylogenetic trees, revealing that CanineCV’s detected in Thailand clustered with CanineCV UCD3-478 for both Cap and Rep genes. However, the phylogenetic trees obtained revealed discrepant patterns among genes by showing distinct and divergent homologous clade of the Rep gene, compared to the Cap gene (Fig. 2B,C).

**Figure 3:**
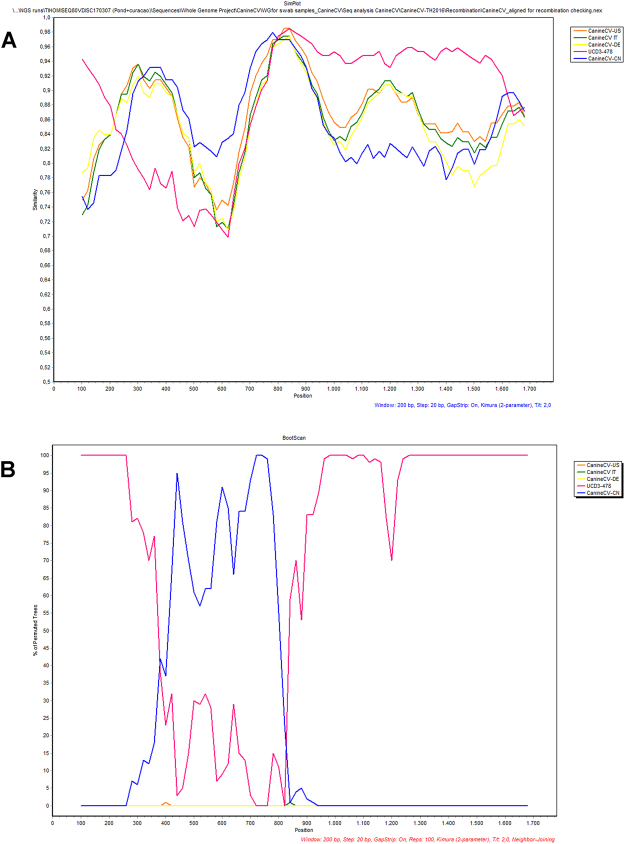
Similarity plot (**A**) and Bootscan analysis (**B**) of CanineCV Thai strain (query), compared with published CanineCV in various strains, showing potential recombination events locating in the Rep gene. The groups consisted of CanineCV strain UCD3-478 (UCD3-478, accession no. KC241983), three groups of reference sequences consisted of CanineCV-US, n = 3 (accession nos. KC241982, KC241984 and JQ821392), CanineCV-IT, n = 18 (accession nos. KT734812-KT734828 and KJ530972), CanineCV-DE, n = 2 (accession nos. KF887949 and KT283604) and CanineCV-CN, n = 1 (strain JZ98/2014, accession no. KT946839). Potential recombinants, CanineCV/TH2016, n = 8; CanineCV_14P105D/TH2016, 14P112N/TH2016, 15P061D/TH2016, CP28/TH2016, CP134/TH2016, CP144/TH2016, CP182/TH2016, CP188TH/2016 were used as a query.

All of the CanineCV’s-TH/2016, except CanineCV CP191_TH/2016, were identified as recombinants using the RDP4 software. The recombination events in these strains were supported by a number of statistical measurement recombination assays including RDP, GENECONV, BootScan, Maxchi, Chimaera, SiScan, 3Seq and LARD at average *p*-value = 1.42 × 10^−4^. Among these viruses, CanineCV’s UCD3-478 and JZ98/2014 were identified as major and minor parents, respectively. The results obtained from RDP analysis were further analyzed to identify potential recombination breakpoints using SIMPLOT software, which also indicated that CanineCV’s TH/2016 (except the CP191 strain) were recombinants. The similarity generated plot for CanineCV’s TH/2016 revealed that these strains had high nucleotide identity to a CanineCV UCD3-478 (pink line) in the initial Rep gene region and a region of the Cap gene. The CanineCV TH/2016 genomes also showed obvious nucleotide similarity to the CanineCV JZ98/2014 strain (blue line) in the middle portion of Rep gene (Fig. 3A). Furthermore, bootscan analysis confirmed the disparities observed in phylogenetic analyses of CanineCV’s TH/2016. The putative recombination breakpoint of the CanineCV’s TH/2016 that contained sequences derived from CanineCV JZ98/2014 was nt 380 to nt 820, within the Rep gene. CanineCV UCD3-478 served as a parental template for the Cap gene (Fig. 3B).

### Sequence comparison of recombinant CanineCV’s TH/2016 with parental strains

Further investigation of recombination events of CanineCV’s TH/2016 was achieved by differentiation of genetic marker positions between recombinants (CanineCV 14P105D) and their parental lineages (CanineCV_UCD3-478 and CanineCV_JZ98/2014) as shown in Table 2. The genetic markers were identified by nucleotide marker positions starting from nt 383 through nt 807 in the CanineCV 14P105D TH/2016 (green shading), which was derived from CanineCV JZ98/2014. This analysis also confirmed the predicted nucleotide recombination break points as shown in RDP and SIMPLOT analyses and was useful to corroborate the site of genetic recombination. Moreover, CanineCV CP191 TH/2016 was also analyzed and it was shown to be most likely recombined with CanineCV JZ98/2014. However, mutant nucleotides were detected in nt 333, which were thus far only observed previously in two Italian CanineCV strains (AZ4133/1, accession no. KT734815 and AZ4133/2, accession no. KT734827).

**Table 2:** Comparison of nucleotide marker positions between recombinant CanineCV 14P105D TH/2016 and its parenteral CanineCV strains.

Genome Position	UCD3-478	JZ98/2014	14P105D TH/2016	CP191TH/2016
**Rep gene**				
327	A	G	A	G
333	C	G	C	T^a^
339	T	A	T	A
345	A	G	A	G
383	C	A	A	A
384	T	A	A	A
390	G	A	A	A
408	T	C	C	C
414	G	C	C	C
426	C	T	T	T
456	A	C	C	C
525	C	G	G	G
540	G	C	C	C
543	C	T	T	T
692	G	T	T	T
762	A	C	C	T
805	A	G	G	G
807	T	G	G	G
825	T	C	T	T
837	T	C	T	C
1095	G	T	G	T
1096	T	G	T	G
**Cap gene**				
1125	T	G	T	T
1182	G	C	G	C
1305	T	C	T	C
1329	A	T	A	T

^a^Specific nucleotide mutation detected in CanineCV CP191 TH/2016 strain.

## Discussion

In the present study samples from dogs with acute respiratory disease in Thailand, in which three respiratory viruses (CIV, CRCoV, CPIV) had been detected in routine screening, were subjected to additional analyses. Partial CanineCV genome sequences were detected by NGS and PCR of lung and swab samples respectively. However, the CanineCV specific ISH was positive only in macrophages present in tracheobronchial lymph nodes and tonsils of one dog. The demonstration of CanineCV in lungs and other organs by NGS and PCR, in the absence of CanineCV ISH signal outside the lymphoid tissues, was consistent with hematogenous spread of CanineCV’s TH/2016. Similar findings of PCR-positive but ISH-negative tests in CanineCV–infected dogs were also recently documented^8^. Full-length genome was obtained from liver tissue, however upon ISH analysis, only the lymphoid tissue had positive signals.

The detection of partial CanineCV genome sequences by several techniques prompted us to perform molecular analyses of CanineCV genomes generated from the positive dogs. It was shown that these clustered into two distinct lineages. The majority of CanineCV’s in Thailand represented a lineage that also contained the closely related CanineCV UCD3-478 from the USA^8^. However, CanineCV CP191/TH2016 was most closely related to CanineCV JZ98/2014, from China. Surprisingly, pairwise distance analysis of CanineCV’s from Thailand showed at least 10.5% genetic difference from a previously characterized and related virus even though they were all present in the same lineage, thus suggesting the detection of novel strains of CanineCV in this study. Preliminary analysis of CanineCV’s detected in Thailand revealed discordant phylogenetic relationship with other CanineCV’s. For instance, the CanineCV’s TH2016 clustered with CanineCV UCD3-478 when compared by analysis of complete genomes and Cap genes. However, they differentially clustered within a new lineage together with the CanineCV UCD3-478 strain when the phylogenetic tree was based on analysis of the Rep gene. Such paradoxical results indicate recombination events during CanineCV TH/2016 evolution. Therefore, we used boot scanning, phylogenetic incongruity, genetic marker identifications and additional sequence analyses to detect recombination events that may have occurred in circulating CanineCV’s, enabling the identification of recombination breakpoints occurring in putative recombinant CanineCV’s TH/2016. Here, we showed a recombination breakpoint at base positions 380–820, located in the Rep gene, which overlaps the putative ORF3 region. The Rep gene encoded by ORF1, plays a critical role in viral replication^19–21^, whereas ORF2 encodes the immunogenic icosahedral capsid protein^22,23^.

Studies of PCV2 genomes have shown that these viruses undergo homologous recombination events contributing to an increased genetic diversity and that those events are most commonly observed in the Rep gene^15–17,24,25^. However, intra- and inter-genotypic recombination events have also been documented to occur in the Cap gene of other circoviruses^26,27^. Mixed infections are a predisposing factor for homologous recombination. The recombination patterns that are generated can be influenced likewise by different mechanistic factors. These include the *ori* site, secondary structure features, sequence similarity and replication/transcription discongruities among other factors^28,29^. In the present study, the presence of a strain (CanineCV CP191/TH2016) most closely related to CanineCV JZ98/2014, indicates that the strain from which the nt region 380–820 of the recombinant viruses derives, circulates in Thailand. Therefore, we speculate that homologous recombination during a mixed infection has most likely generated the present recombinant strain. However, with the present data, it is not clear which mechanistic factor influenced the occurrence of the discussed recombination event. Moreover, a recent study on PCV2 revealed that the virus may have gradually evolved into several strains due to selective pressure such as vaccination by promoting a change of viral capsid protein away from the vaccine specific antigenic determinants^30,31^. Furthermore, in addition to a recombination breakpoint in CanineCV’s TH/2016, a point mutation (nt 333) in Rep gene (nt 333) was observed in CanineCV-CP191 TH/2016, a mutation observed previously in only two Italian CanineCVs AZ4133-1/13, KT734815 and AZ4133-2/13, KT734827). Although point mutations are a potential source of circovirus evolution as observed in PCV2^27^, the effect of this mutation on CanineCV is unknown.

Analysis of genome sequences of the novel Thai CanineCV’s identified in this study, showed the presence of ORF3, a recently recognized overlapping anti-directional region of ORF1 gene that is dispensable for virus replication^32^ but has been linked to virus-induced apoptosis in PCV2^33^. Recent studies showed that ORF3-deficient PCV2 were less pathogenic in mice in comparison to analogous infections with wild type strains^34^. However, the relevance in natural infections remains questionable as virus attenuation was not observed upon infection of pigs with ORF-3-deficit PCV2^32^. Investigations into the function of the ORF-3 region in addition to phenotypic consequences of any mutations in this region of the Thai CanineCV’s should therefore be undertaken. The recombination breakpoint of CanineCV strains in our study occurred in the Rep gene, which also encompasses the ORF3 region. It is not clear if this recombination event impacts on the pathogenesis of CanineCV infection, calling for further investigation.

In summary we present the first comprehensive description of circulating CanineCVs in Thailand and document a recombination event that adds to our understanding of the genetic diversity of recently recognized CanineCV’s. Retrospective studies on historical samples taken from CanineCV-infected dogs may provide answers as to when such a recombinant virus first emerged. The identification of such genetic recombination events among CanineCV’s also supports whole genome sequencing as an alternative to phylogenetic analysis based on only single genome regions. Further applied studies should be undertaken into the functioning of CanineCV proteins, to better understand how mutations and/or recombination events alter CanineCV pathogenesis and transmission.

## Materials and Methods

### Animal description and sample collection

Fresh tissue samples were collected from brain, lung, liver, kidneys, tonsil and tracheobronchial lymph nodes of three dogs (14P105D, 14P112N, 15P061D) with respiratory symptoms in Bangkok, Thailand, that were also subjected to routine post-mortem gross and histopathological examination at Department of Pathology, Faculty of Veterinary Science, Chulalongkorn University, Bangkok, Thailand. In addition, twenty nasal or oral swabs were collected from other Thai dogs showing various degrees of respiratory problems (Table 1), according to approval of Chulalongkorn University Animal Care and Use Committee (No. 1431005). All methods were performed in accordance guidelines and regulations.

### Routine virological testing

Extracted nucleic acids from the fresh tissues and swabs were subjected to routine laboratory investigations. Common viruses of canine infectious respiratory disease complex (CIRDC) including canine influenza virus (CIV), canine parainfluenza virus (CPIV), canine distemper virus, (CDV), canine respiratory coronavirus (CRCoV), canine adenovirus type 1 and 2 (CAdV1-2) and canine herpesvirus type 1 (CaHV-1) were screened for by multiplex PCRs^35,36^. Pan-PCRs using degenerate primers specific for paramyxoviruses, coronaviruses and herpesviruses were also performed on extracted nucleic acids as described previously^37–39^ with some modifications. A commercial rapid CPV/CCV Ag test kit (Bionote Inc., Suwon, South Korea), was used to test intestinal swabs taken from the three necropsied dogs, to test for the presence of canine parvovirus (CPV) and canine enteric coronavirus (CCV) infections.

### Sequence independent single primer amplification and next-generation sequencing

Fresh lung samples of dogs No. 14P105D and No. 14P112N were prepared for NGS using a modified sequence-independent single-primer amplification (SISPA) protocol as previously described^40^. Briefly, after homogenization, RNA was extracted by QIAmp Viral RNA mini kit (Qiagen, Hilden, Germany), cDNA was constructed by using a mixture of random and non-ribosomal hexamers^41,42^ with SuperScript IV (Invitrogen, Thermo Fisher Scientific, Waltham, MA, USA), followed by Klenow reaction. Next, Taq polymerase (Invitrogen, Thermo Fisher Scientific, Waltham, MA, USA) was used to randomly amplify from cDNA. The PCR product was purified using Monarch^®^ PCR and DNA clean up kit (New England Biolab, Frankfurt, Germany). Final DNA concentrations were measured by PicoGreen (Invitrogen, Thermo Fisher Scientific, Waltham, MA, USA). A DNA Library was then constructed following the Nextera XT protocol (Illumina, USA). Samples were then deep sequenced on an Illumina MiSeq system using MiSeq Reagent kit V3 (300 × 2 cycles). Raw reads were initially screened using an *in-house* metagenomics pipeline to identify interesting viral reads^40^. Reference assembly was performed with CLC Genomics Workbench 9.0. Phylogenetic analyses were carried out using MEGA 7.

### Genome sequencing and CanineCV-specific PCR

PCRs with Sanger sequencing were performed to confirm the presence of specific viral sequences. CanineCV genomes were obtained by PCR amplification using degenerated primers designed from various strains of published CanineCV genome in GenBank (Supplementary Table S2). Briefly, PCR reactions comprised of a mixture of 1 U of Phusion DNA polymerase (New England Biolab, UK), 10 mM of dNTP in 5× Phusion HF Buffer, 10 *µ*M final concentration of each primer and 2 *µl* of template. Cycler conditions consisted of initial denaturation for 98 °C for 30 sec, followed by 45 cycles of 98 °C, 20 sec, 50 °C, 30 sec, 72 °C, 1 min and 72 °C, 7 min for final extension. The PCR product was run on a 1% agarose gel and then purified using Monarch DNA Gel extraction kit (New England Biolab, Frankfurt, Germany). All the purified PCR products were subjected to Sanger sequencing in order to determine the complete nucleotide sequences of detected CanineCV. The resulting sequences of CanineCV were deposited in GenBank.

### CanineCV in canine respiratory swab samples

The genetic diversity of CanineCV’s was investigated in the lung tissue sample from dog no. 15P061D and 20 respiratory swabs, using specific CanineCV PCR amplification and sequencing. Primers were designed based on CanineCV strains available in GenBank, targeting 517 bp of partial capsid gene (Supplementary Table S2). Subsequently, a panel of primers designed for amplification of the CanineCV genome was used to further analyze samples which were CanineCV positive (Supplementary Table S2). The PCR reaction consisted of 1 U of Phusion DNA polymerase (New England Biolab, UK), 10 mM of dNTP in 5x Phusion HF Buffer, 10 *µ*M final concentration of each primer and 2 *µl* of cDNA. Cycler conditions consisted of initial denaturation for 98 °C for 30 sec, followed by 45 cycles of 98 °C, 20 sec, 50 °C, 30 sec, 72 °C, 1 min and 72 °C, 7 min for final extension. CanineCV positive PCR products were visualized on 1% Agarose gel and then purified by Monarch DNA Gel extraction kit (New England Biolab, Frankfurt, Germany) prior to Sanger sequencing (Eurofins Genomics).

### Sequence analysis and genomic recombination

CanineCV sequences from all positive samples were aligned using MAFFT alignment version 7 (http://mafft.cbrc.jp/alignment/server/) and MEGA 7 based on nucleotide sequences of the nearly complete genome (nt 49 to 1949), replicase, and capsid genes. These alignments were used to construct phylogenetic trees using maximum likelihood method with TN93+G for complete genome, K2+G model for Rep gene and HKY+G for Cap gene as best-fit model of nucleotide substitution according to Bayesian Information Criterion^40^. All phylogenetic trees were tested by bootstrapping with 1000 replicates. Sequence pairwise distances of CanineCV genomes were calculated using Maximum Composite Likelihood model^43^. The evolutionary analyses were conducted in MEGA 7^44^. In order to identify the possibly recombinant origin(s) of CanineCV TH/2016 strains, a recombination detection program 4 (RDP4) package v. Beta 4.94, an integrated software program^45^ was used. Briefly, the recombination events of CanineCV’s TH/2016 were detected by using a range of recombination detection methods including RDP, GeneConv, Bootscan, MaxChi, Chimera, SiScan and 3Seq with cut-off acceptable *p*-value at 0.01. Bonferroni correction was used as a general setting. The complete genomes of CanineCV’s available in GenBank were included in the RDP analyses. Similarity plot and bootscan analysis in SIMPLOT software package version 3.5.1^46^ were also performed on CanineCV sequences. Briefly, a bootscaning analysis was performed on four groups of complete CanineCV genome sequences based on differential clustering in the phylogenetic tree. The bootscan analysis was tested under the Kimura-2 parameter (K2P) model for 100 replications, with a window size of 200 bp, step size of 20 bp and a transition/transversion ratio of 2. The similarity plot was performed under the same group and conditions with GapStrip model. Identified recombinant sequences were also allegorized with their potential parents by genetic marker positions to obtain further evidence of genetic recombination.

### Molecular detection of CanineCV in canine tissues

Primers targeting the capsid gene of CanineCV were used to assess the distribution of this virus in the dogs. In addition to lung tissue, brain, liver, tracheobronchial lymph node, tonsil and kidneys were analyzed for the presence of CanineCV (Supplementary Table S2). Briefly, the extracted nucleic acids from each sample were used as template for cDNA synthesis as described above. The PCR reaction consisted of 5x OneTaq Buffer, 10 mM of dNTP, 1 U Hot start Taq polymerase (New England Biolab, Ipswich, MA, U.S.A.), 10 µM final concentration of each primer and 2 *µl* of cDNA. Cycler conditions consisted of initial denaturation for 94 °C for 5 min, followed by 45 cycles of 94 °C, 30 sec, 50 °C, 30 sec, 72 °C, 1 min and 72 °C, 7 min for final extension. CanineCV positive PCR products were visualized on 1% Agarose gel and then purified by Monarch DNA Gel extraction kit (New England Biolab, Frankfurt, Germany) prior to Sanger sequencing (Eurofins Genomics). To confirm the presence of CanineCV, *in situ*-hybridization (ISH) was performed on FFPE tissues, including lymph nodes, tonsils, thymus, spleen, pancreas, small intestine, kidney, urinary bladder, heart, trachea and lungs. ISH was performed as previously described with minor variations^47^. Probes were designed from alignment of published CanineCV genomes as follows: sense Probe: 5′-ACTACTGAAAGATAAAGGCCTCTCG-3′, antisense Probe: 5′CGAGAGGCCTTTATCTTTCAGTAGT-3′, and labelled with Digoxigenin (DIG) that covered 25 bp of the CanineCV (nt 905–929). Briefly, after de-parafinization of FFPE sections and hydration in graded ethanol, samples were washed in diethyl pyrocarbonate (DEPC)-treated water. Proteolytic digestion of samples was performed with 1 µg/ml proteinase K (Roche Diagnostics, Basel, Switzerland). An overnight incubation at 52 °C with 1000 ng/100 µl sense and antisense probe, respectively, was performed following post-fixation, acetylation and pre-hybridization. Detection was carried out using an anti-DIG antibody (diluted 1:200) conjugated with alkaline phosphatase (Roche Diagnostics, Basel, Switzerland) in combination with nitrobluetetrazoliumchloride (NBT, Sigma-Aldrich, St. Louis, MO, U.S.A.) and 5-bromo-4-chloro-3-indolyl phosphatate (BCIP, X-Phosphate, Sigma-Aldrich, St. Louis, MO, U.S.A.) as substrates. Distinct, purple precipitates, that were clearly cellular located, were considered positive.

## Electronic supplementary material

Supplementary information
