# Supplementary material for: Novel canine circovirus strains from Thailand: Evidence for genetic recombination
Source: Sci Rep. 2018 May 14;8:7524. doi: 10.1038/s41598-018-25936-1 (PMC5951951; doi:10.1038/s41598-018-25936-1)
Supplement: Supplementary file 1 — Supplementary information [file 41598_2018_25936_MOESM1_ESM.doc]

**Novel canine circovirus strains from Thailand: Evidence for genetic recombination**

Chutchai Piewbang^1,2^, Wendy K. Jo^2^, Christina Puff^3^, Erhard van der Vries^2^, Sawang Kesdangsakonwut^1^, Anudep Rungsipipat^1^, Jochen Kruppa^4^, Klaus Jung^4^, Wolfgang Baumgärtner^3^, Somporn Techangamsuwan^1,5^, Martin Ludlow^2^, Albert D.M.E. Osterhaus^2*^

^1^ Department of Pathology, Faculty of Veterinary Science, Chulalongkorn University, Pathumwan, Bangkok 10330, Thailand

^2^ Research Center for Emerging Infections and Zoonoses, University of Veterinary Medicine (TiHo-RIZ), Bünteweg 17, Hannover 30559, Germany

^3^ Department of Pathology, University of Veterinary Medicine, Bünteweg 17, Hannover 30559, Germany

^4^ Institute for Animal Breeding and Genetics, University of Veterinary Medicine, Bünteweg 17p, Hannover 30559, Germany

^5^ STAR Diagnosis and Monitoring of Animal Pathogen, Faculty of Veterinary Science, Chulalongkorn University, Pathumwan, Bangkok 10330, Thailand

**^*^Corresponding author**: Prof. Albert Osterhaus.

Research Center for Emerging Infections and Zoonoses, University of Veterinary Medicine, Hannover 30559, Germany (TiHo-RIZ).

Tel: +49 511 953 6140

Fax: +49 511 953 826140

Email address: albert.osterhaus@tiho-hannover.de

**Supplementary table 1. Pairwise distances of CanineCV TH/2016 genomes compared with other published CanineCV strains.**

| **Sequences** | **1)** | **2)** | **3)** | **4)** | **5)** | **6)** | **7)** | **8)** | **9)** | **10)** | **11)** | **12)** | **13)** | **14)** | **15)** | **16)** | **17)** | **18)** | **19)** | **20)** | **21)** | **22)** | **23)** | **24)** | **25)** | **26)** | **27)** | **28)** | **29)** | **30)** | **31)** | **32)** | **33)** | **34)** |
| --- | --- | --- | --- | --- | --- | --- | --- | --- | --- | --- | --- | --- | --- | --- | --- | --- | --- | --- | --- | --- | --- | --- | --- | --- | --- | --- | --- | --- | --- | --- | --- | --- | --- | --- |
| 1. **KC241982.1** |  |  |  |  |  |  |  |  |  |  |  |  |  |  |  |  |  |  |  |  |  |  |  |  |  |  |  |  |  |  |  |  |  |  |
| 1. **KC241984.1** | 0.054 |  |  |  |  |  |  |  |  |  |  |  |  |  |  |  |  |  |  |  |  |  |  |  |  |  |  |  |  |  |  |  |  |  |
| 1. **KJ530972.1** | 0.054 | 0.022 |  |  |  |  |  |  |  |  |  |  |  |  |  |  |  |  |  |  |  |  |  |  |  |  |  |  |  |  |  |  |  |  |
| 1. **KT734815.1** | 0.054 | 0.023 | 0.013 |  |  |  |  |  |  |  |  |  |  |  |  |  |  |  |  |  |  |  |  |  |  |  |  |  |  |  |  |  |  |  |
| 1. **KT734827.1** | 0.054 | 0.023 | 0.013 | 0.000 |  |  |  |  |  |  |  |  |  |  |  |  |  |  |  |  |  |  |  |  |  |  |  |  |  |  |  |  |  |  |
| 1. **KT734814.1** | 0.052 | 0.024 | 0.018 | 0.019 | 0.019 |  |  |  |  |  |  |  |  |  |  |  |  |  |  |  |  |  |  |  |  |  |  |  |  |  |  |  |  |  |
| 1. **KT734820.1** | 0.049 | 0.023 | 0.019 | 0.020 | 0.020 | 0.016 |  |  |  |  |  |  |  |  |  |  |  |  |  |  |  |  |  |  |  |  |  |  |  |  |  |  |  |  |
| 1. **KT734824.1** | 0.049 | 0.023 | 0.019 | 0.020 | 0.020 | 0.016 | 0.000 |  |  |  |  |  |  |  |  |  |  |  |  |  |  |  |  |  |  |  |  |  |  |  |  |  |  |  |
| 1. **KT734817.1** | 0.054 | 0.029 | 0.020 | 0.024 | 0.024 | 0.014 | 0.020 | 0.020 |  |  |  |  |  |  |  |  |  |  |  |  |  |  |  |  |  |  |  |  |  |  |  |  |  |  |
| 1. **KT734818.1** | 0.055 | 0.030 | 0.021 | 0.025 | 0.025 | 0.015 | 0.020 | 0.020 | 0.001 |  |  |  |  |  |  |  |  |  |  |  |  |  |  |  |  |  |  |  |  |  |  |  |  |  |
| 1. **KT734819.1** | 0.057 | 0.025 | 0.020 | 0.022 | 0.022 | 0.019 | 0.017 | 0.017 | 0.018 | 0.019 |  |  |  |  |  |  |  |  |  |  |  |  |  |  |  |  |  |  |  |  |  |  |  |  |
| 1. **KT734822.1** | 0.056 | 0.025 | 0.020 | 0.020 | 0.020 | 0.020 | 0.019 | 0.019 | 0.023 | 0.024 | 0.020 |  |  |  |  |  |  |  |  |  |  |  |  |  |  |  |  |  |  |  |  |  |  |  |
| 1. **KT283604.1** | 0.060 | 0.036 | 0.034 | 0.033 | 0.033 | 0.034 | 0.032 | 0.032 | 0.033 | 0.034 | 0.033 | 0.034 |  |  |  |  |  |  |  |  |  |  |  |  |  |  |  |  |  |  |  |  |  |  |
| 1. **KT734813.1** | 0.049 | 0.030 | 0.031 | 0.031 | 0.031 | 0.034 | 0.030 | 0.030 | 0.033 | 0.034 | 0.036 | 0.031 | 0.018 |  |  |  |  |  |  |  |  |  |  |  |  |  |  |  |  |  |  |  |  |  |
| 1. **KT734816.1** | 0.058 | 0.030 | 0.027 | 0.025 | 0.025 | 0.030 | 0.027 | 0.027 | 0.033 | 0.033 | 0.029 | 0.029 | 0.018 | 0.016 |  |  |  |  |  |  |  |  |  |  |  |  |  |  |  |  |  |  |  |  |
| 1. **KF887949.1** | 0.057 | 0.029 | 0.029 | 0.027 | 0.027 | 0.032 | 0.029 | 0.029 | 0.036 | 0.036 | 0.032 | 0.031 | 0.020 | 0.020 | 0.017 |  |  |  |  |  |  |  |  |  |  |  |  |  |  |  |  |  |  |  |
| 1. **KT734812.1** | 0.039 | 0.039 | 0.043 | 0.043 | 0.043 | 0.041 | 0.039 | 0.039 | 0.043 | 0.043 | 0.044 | 0.043 | 0.043 | 0.037 | 0.044 | 0.046 |  |  |  |  |  |  |  |  |  |  |  |  |  |  |  |  |  |  |
| 1. **KT734828.1** | 0.039 | 0.039 | 0.043 | 0.043 | 0.043 | 0.041 | 0.039 | 0.039 | 0.043 | 0.043 | 0.044 | 0.043 | 0.043 | 0.037 | 0.044 | 0.046 | 0.000 |  |  |  |  |  |  |  |  |  |  |  |  |  |  |  |  |  |
| 1. **KT734823.1** | 0.041 | 0.042 | 0.044 | 0.046 | 0.046 | 0.043 | 0.041 | 0.041 | 0.046 | 0.046 | 0.043 | 0.046 | 0.048 | 0.040 | 0.047 | 0.048 | 0.009 | 0.009 |  |  |  |  |  |  |  |  |  |  |  |  |  |  |  |  |
| 1. **KT734826.1** | 0.041 | 0.042 | 0.044 | 0.046 | 0.046 | 0.043 | 0.041 | 0.041 | 0.046 | 0.046 | 0.043 | 0.046 | 0.048 | 0.040 | 0.047 | 0.048 | 0.009 | 0.009 | 0.000 |  |  |  |  |  |  |  |  |  |  |  |  |  |  |  |
| 1. **JQ821392.1** | 0.040 | 0.043 | 0.043 | 0.041 | 0.041 | 0.041 | 0.040 | 0.040 | 0.042 | 0.043 | 0.041 | 0.044 | 0.049 | 0.040 | 0.044 | 0.046 | 0.032 | 0.032 | 0.034 | 0.034 |  |  |  |  |  |  |  |  |  |  |  |  |  |  |
| 1. **KT734821.1** | 0.063 | 0.034 | 0.036 | 0.034 | 0.034 | 0.037 | 0.035 | 0.035 | 0.040 | 0.041 | 0.036 | 0.037 | 0.037 | 0.034 | 0.029 | 0.029 | 0.052 | 0.052 | 0.054 | 0.054 | 0.051 |  |  |  |  |  |  |  |  |  |  |  |  |  |
| 1. **KT734825.1** | 0.063 | 0.034 | 0.036 | 0.034 | 0.034 | 0.037 | 0.035 | 0.035 | 0.040 | 0.041 | 0.036 | 0.037 | 0.037 | 0.034 | 0.029 | 0.029 | 0.052 | 0.052 | 0.054 | 0.054 | 0.051 | 0.000 |  |  |  |  |  |  |  |  |  |  |  |  |
| 1. **KT946839.1** | 0.120 | 0.116 | 0.116 | 0.115 | 0.115 | 0.117 | 0.117 | 0.117 | 0.117 | 0.118 | 0.118 | 0.119 | 0.120 | 0.112 | 0.115 | 0.117 | 0.117 | 0.117 | 0.117 | 0.117 | 0.113 | 0.118 | 0.118 |  |  |  |  |  |  |  |  |  |  |  |
| 1. **KC241983.1** | 0.147 | 0.166 | 0.174 | 0.173 | 0.173 | 0.174 | 0.169 | 0.169 | 0.171 | 0.172 | 0.177 | 0.173 | 0.037 | 0.170 | 0.168 | 0.171 | 0.173 | 0.173 | 0.177 | 0.177 | 0.169 | 0.164 | 0.164 | 0.175 |  |  |  |  |  |  |  |  |  |  |
| 1. **14P105D** | 0.121 | 0.142 | 0.147 | 0.147 | 0.147 | 0.147 | 0.143 | 0.143 | 0.149 | 0.150 | 0.153 | 0.148 | 0.145 | 0.138 | 0.144 | 0.145 | 0.145 | 0.145 | 0.147 | 0.147 | 0.142 | 0.137 | 0.137 | 0.143 | 0.105 |  |  |  |  |  |  |  |  |  |
| 1. **14P112N** | 0.130 | 0.150 | 0.157 | 0.156 | 0.156 | 0.155 | 0.152 | 0.152 | 0.158 | 0.159 | 0.162 | 0.157 | 0.154 | 0.148 | 0.153 | 0.154 | 0.155 | 0.155 | 0.156 | 0.156 | 0.151 | 0.145 | 0.145 | 0.152 | 0.114 | 0.008 |  |  |  |  |  |  |  |  |
| 1. **15P061D** | 0.126 | 0.146 | 0.151 | 0.150 | 0.150 | 0.150 | 0.146 | 0.146 | 0.154 | 0.154 | 0.157 | 0.152 | 0.149 | 0.143 | 0.148 | 0.148 | 0.149 | 0.149 | 0.150 | 0.150 | 0.147 | 0.141 | 0.141 | 0.147 | 0.111 | 0.005 | 0.014 |  |  |  |  |  |  |  |
| 1. **CP28** | 0.122 | 0.145 | 0.150 | 0.149 | 0.149 | 0.148 | 0.146 | 0.146 | 0.151 | 0.152 | 0.156 | 0.151 | 0.147 | 0.141 | 0.147 | 0.147 | 0.146 | 0.146 | 0.147 | 0.147 | 0.143 | 0.139 | 0.139 | 0.144 | 0.107 | 0.002 | 0.011 | 0.007 |  |  |  |  |  |  |
| 1. **CP134** | 0.123 | 0.145 | 0.150 | 0.149 | 0.149 | 0.147 | 0.145 | 0.145 | 0.151 | 0.152 | 0.156 | 0.151 | 0.147 | 0.141 | 0.147 | 0.147 | 0.145 | 0.145 | 0.147 | 0.147 | 0.143 | 0.139 | 0.139 | 0.146 | 0.110 | 0.005 | 0.014 | 0.006 | 0.006 |  |  |  |  |  |
| 1. **CP144** | 0.122 | 0.144 | 0.149 | 0.149 | 0.149 | 0.146 | 0.145 | 0.145 | 0.150 | 0.151 | 0.155 | 0.150 | 0.147 | 0.140 | 0.146 | 0.147 | 0.146 | 0.146 | 0.147 | 0.147 | 0.143 | 0.139 | 0.139 | 0.144 | 0.107 | 0.002 | 0.010 | 0.006 | 0.003 | 0.003 |  |  |  |  |
| 1. **CP182** | 0.122 | 0.143 | 0.149 | 0.148 | 0.148 | 0.147 | 0.145 | 0.145 | 0.151 | 0.151 | 0.154 | 0.149 | 0.146 | 0.140 | 0.145 | 0.146 | 0.147 | 0.147 | 0.148 | 0.148 | 0.143 | 0.133 | 0.133 | 0.145 | 0.106 | 0.001 | 0.010 | 0.005 | 0.003 | 0.004 | 0.001 |  |  |  |
| 1. **CP188** | 0.122 | 0.143 | 0.149 | 0.148 | 0.148 | 0.147 | 0.145 | 0.145 | 0.151 | 0.151 | 0.154 | 0.149 | 0.146 | 0.140 | 0.145 | 0.146 | 0.147 | 0.147 | 0.148 | 0.148 | 0.143 | 0.133 | 0.133 | 0.145 | 0.106 | 0.001 | 0.010 | 0.005 | 0.003 | 0.004 | 0.001 | 0.000 |  |  |
| 1. **CP191** | 0.119 | 0.113 | 0.113 | 0.111 | 0.111 | 0.114 | 0.112 | 0.112 | 0.117 | 0.118 | 0.116 | 0.117 | 0.116 | 0.111 | 0.112 | 0.115 | 0.115 | 0.115 | 0.116 | 0.116 | 0.114 | 0.117 | 0.117 | 0.039 | 0.173 | 0.134 | 0.142 | 0.138 | 0.134 | 0.137 | 0.135 | 0.135 | 0.135 | 0.000 |

**Supplementary table 2.** Sequences of CanineCV specific primers used for complete genome.

| **Genome position** | **Sequence (5’-3’)** | **Direction** | **Product size**  **(bp)** |
| --- | --- | --- | --- |
| **121-140** | GCGCCGGACGCTAAGTACTT | Forward | 968 |
| **1088-1067** | GTCACGWGTGTTTATTRGYYGG | Reverse |  |
| **605-626** | AATGGTGGGAYGGYTACGATGG^a^ | Forward | 437 |
| **1041-1021** | AAGGGGGGTGAACAGGTAAAC^a^ | Reverse |  |
| **1022-1044** | TTTACCTGTTCACCCCCCTTCGA^b^ | Forward | 517 |
| **1538-1515** | GGAAGAGGYAATGCTACAAGATCA^b^ | Reverse |  |
| **1306-1324** | GTKCCTCTKGTYAGCCATG | Forward | 741 |
| **2046-2028** | GTGCTGTGTCTGTGACGAG | Reverse |  |

^a^Primers used for filling the gap of CanineCV genome, which achieved from NGS read.

^b^Primers used for complete CanineCV whole genome and for CanineCV-specific PCR targeting capsid gene region.
